# Cardiopulmonary interactions in left heart failure

**DOI:** 10.3389/fphys.2023.1237741

**Published:** 2023-08-08

**Authors:** Andrea C. Alvarado, Michael R. Pinsky

**Affiliations:** Department of Critical Care Medicine, University of Pittsburgh, Pittsburgh, PA, United States

**Keywords:** left heart failure, cardiopulmonary interaction, heart lung interactions, COPD and left heart failure, OSA and left heart failure

## Abstract

The primary impact of ventilation and ventilatory efforts on left ventricular (LV) function in left ventricular dysfunction relate to how changes in intrathoracic pressure (ITP) alter the pressure gradients for venous return into the chest and LV ejection out of the chest. Spontaneous inspiratory efforts by decreasing ITP increase both of these pressure gradients increasing venous blood flow and impeding LV ejection resulting in increased intrathoracic blood volume. In severe heart failure states when lung compliance is reduced, or airway resistance is increased these negative swings in ITP can be exacerbated leading to LV failure and acute cardiogenic pulmonary edema. By merely reversing these negative swings in ITP by the use of non-invasive continuous positive airway pressure (CPAP), these profoundly detrimental forces can be immediately reversed, and cardiovascular stability can be restored in moments. This forms the clinical rationale for the immediate use of CPAP for the treatment of acute cardiogenic pulmonary edema. Increasing ITP during positive pressure ventilation decreases the pressure gradients for venous return and LV ejection decreasing intrathoracic blood volume. In a hypovolemic patient even with LV dysfunction this can result in hypotension due to inadequate LV preload. Minor increases in ITP as occur using pressure-limited positive-pressure ventilation primarily reverse the increased LV afterload of negative swings in ITP and if fluid overload was already present, minimally alter cardiac output. The effect of changes in lung volume on LV function are related primarily to its effects on right ventricular (RV) function through changes in pulmonary vascular resistance and overdistention (hyperinflation). In acute lung injury with alveolar collapse, positive pressure ventilation may reduce pulmonary vascular resistance if alveolar recruitment predominates. Hyperinflation, however, impedes diastolic filling while simultaneously increasing pulmonary vascular resistance. Thus, increasing lung volume can reduce RV afterload by reversing hypoxic pulmonary vasoconstriction or increase afterload by overdistention. Hyperinflation can also impede RV filling. All of these processes can be readily identified at the bedside using echocardiography.

## Introduction

**FIGURE 1:**
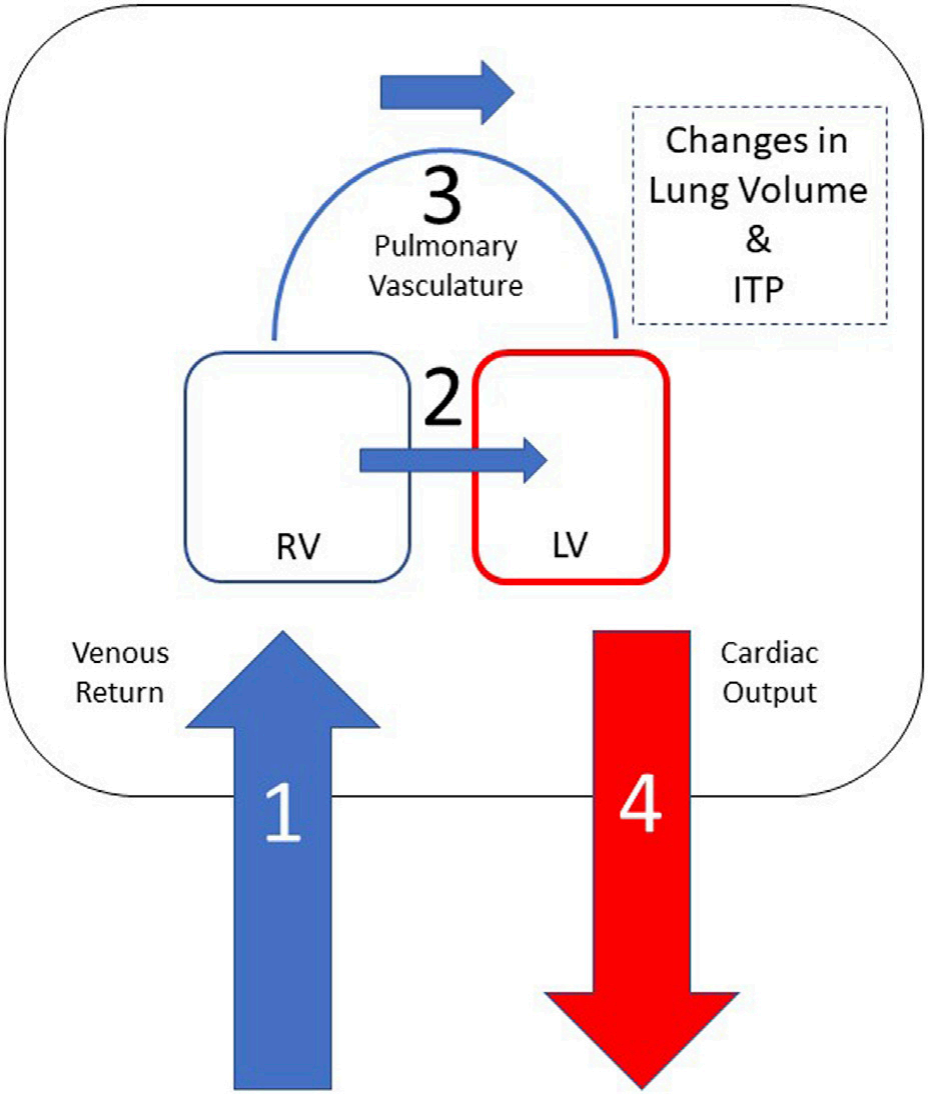
Heart-lung interactions can be simplified into 4 primary processes based on this schematic representation of the heart and thoracic compartment. Positive intrathoracic pressure leads to a decrease in venous return (1) and, consequently, decreased right ventricular preload. Positive ITP also leads to a decreased left ventricular afterload, which results in improved LV ejection and improved cardiac output (4). Negative swings in intrathoracic pressure lead to increase in venous return (1), which causes right ventricular dilation and shift the intraventricular septum towards the left (2) leading to decreased left ventricular end diastolic filling and, consequently, decreased cardiac output (4). Lung hyperinflation leads to increased pulmonary vascular resistance (3), which increases right ventricular afterload and, in volume overloaded states, cause RV dilation leading to a shift of the intraventricular septum towards the left (2) decreasing left ventricular end diastolic filling and, consequently, decreased left ventricular stroke volume (4).

Understanding the hemodynamic coupling of the heart and lungs plays a significant role in managing patients in the critical care setting. The basic heart-lung physiology has been discovered earlier in the collection of papers. Understanding diastolic and systolic left heart failure is of upmost importance when treating patients with a variety of other common medical conditions such as sleep apnea, ARDS and COPD as these conditions are deeply intertwined. These interactions are magnified in the setting of decreased left ventricular (LV) function. Here we seek to explain how clinical presentations affect the left ventricle and how management of these conditions can have a profound effect on LV function. For simplistic sake, the forces that cause clinically relevant heart-lung interactions are illustrated in Figure 1.

There are multiple aspects of cardiopulmonary physiology that affect patients with heart failure. Lung volume and hyperinflation affect autonomic tone and pulmonary vascular resistance. In 1966 de Burgh Daly et al. showed that the correlation between systemic vascular resistance and ventilation was secondary to reflexes from intrapulmonary receptors altered by changes in lung volume. Lung inflation by either negative or positive intrathoracic pressure (ITP), in the setting of blocked carotid sinus and aortic arch baroreceptors, led to an immediate decrease in systemic vasomotor tone (De Burgh Daly et al., 1967). Furthermore, hyperinflation increases pulmonary vascular resistance by compressing alveolar vessels and impedes biventricular filling by increasing juxtacardiac ITP. Both processes impede right ventricular (RV) ejection and LV filling by decreasing LV diastolic compliance and reducing pulmonary venous blood flow, respectively. Changes in ITP independent of changes in lung volume also profoundly impact LV function. Spontaneous ventilatory efforts create negative swings in ITP that lead to decreasing right atrial pressure, increasing intraabdominal pressure by diaphragmatic contraction, increasing mean systemic pressure thereby increasing the pressure gradient for venous return to the heart while simultaneously impeding LV ejection by increasing LV afterload due to the increase in LV transmural ejection pressure (aortic pressure minus ITP). In 1979, Buda et al. used Mueller and Valsalva maneuvers to produce negative and positive intrathoracic pressure, respectively, in awake humans. They demonstrated that sustained negative ITP caused by a Mueller maneuver immediately increased LV end systolic volume as a function of the increased LV ejection pressure. Thus, spontaneous inspiration-associated decreases in ITP will increase LV afterload, and as the negative ITP swings become from pronounced, as may occur with increases airway resistance (asthma), decreased lung compliance (acute respiratory distress syndrome) or an occluded airway (obstructed breathing), LV systolic function will become more impaired and can result in acute cardiogenic pulmonary edema and, if sustained, chronic LV failure (Buda et al., 1979). On the other hand, positive pressure ventilation decreases right ventricular (RV) preload (i.e., decreased venous return) and may alter RV afterload by modifying pulmonary vascular resistance through a variety of mechanisms. Positive pressure ventilation also decreases left ventricular afterload to the extent that ITP also increases, but this effect is less important than the decrease in venous return. However, if positive pressure ventilation is used to eliminate large negative swings in ITP, such as those seen with vigorous inspiratory efforts in patients with asthma and obstructive breathing, then the reduced LV afterload can improve LV ejection (Pinsky et al., 1985). Collectively, these studies showed that positive and negative ITP have a significant effect on cardiac function in patients with heart failure. Given the coupled effect of changes in ITP and lung volume on RV filling, RV ejection and ventricular interdependence, it is clear that ventilatory maneuvers and pulmonary disease can be profoundly deleterious for a variety of reasons (Feihl and Broccard, 2009; Grübler et al., 2017; Cundrle et al., 2019). For example, while negative swings in ITP during forced spontaneous inspiratory efforts will cause right atrial pressure to become sub-atmospheric, the associated increase in venous return to the RV is limited as the large venous conduits collapse as they enter the thorax. Further decreases in ITP will not increase venous return further owing to this flow limitation. However, no such limitation exists for the effects of negative swings in ITP on LV ejection. Thus, progressively more negative swings in ITP will selectively increase LV afterload impeding LV ejection and promoting LV dilation, pulmonary venous congestion and pulmonary edema.

Here we will discuss the importance of heart-lung interactions in various clinical scenarios including acute cardiogenic pulmonary edema (ACPE), ventilatory weaning, chronic obstructive pulmonary disease (COPD), obstructive sleep apnea (OSA), and acute respiratory distress syndrome (ARDS).

### Acute cardiogenic pulmonary edema

Left heart failure can be characterized by systolic dysfunction, diastolic dysfunction, or both. In developed countries, systolic ventricular failure is most commonly associated with ischemia (i.e., myocardial infarction) and hypertension, which also contributes to diastolic dysfunction. In other parts of the world, Chagas disease and rheumatic heart disease are more prevalent causes. Understanding heart lung interactions is essential for the management of all left heart failure patients. Negative intrathoracic pressure leads to increased LV transmural pressure increasing LV afterload. In patients with left heart failure, negative swings in intrathoracic pressure can markedly decrease LV ejection leading to rapid increases in LV filling pressure and secondary (hydrostatic) pulmonary edema. The spontaneous breathing efforts also represent a metabolic stress on the body. During periods of increased work of breathing, spontaneous ventilation increases oxygen demand of respiratory muscles as well as of the myocardium and may induce circulatory insufficiency and visceral organ ischemia (McMurray and Pfeffer, 2005; Gomez et al., 2013; Tavazzi, 2021).

Based on these principles the primary treatment of acute cardiogenic pulmonary edema should be the immediate abolishment of negative swings in ITP. While hypovolemic patients may decrease their cardiac output when positive pressure ventilation is initiated due to the decrease in preload (moving leftward on the ventricular function curve), these effects are minimized if continuous positive airway pressure (CPAP) or bilevel positive airway pressure (BiPAP) are used in patients with acute heart failure, because the spontaneous inspiratory efforts remain but the negative swings in ITP are markedly diminished or abolished. Thus, CPAP and BiPAP selectively reduce LV afterload shifting the ventricular function curve upward in patients with acute heart failure without decreasing venous return. Numerous clinical trials have shown the immediate beneficial cardiovascular effects of initiating CPAP or BiPAP. These findings were reinforced by a meta-analysis by Weng et al. showing less need for endotracheal intubation and a trend toward reduced mortality when non-invasive ventilation is initiated on patients presenting with acute cardiogenic pulmonary edema (Weng et al., 2010). Unfortunately, hyperinflation will impede venous return by increasing ITP. Although LV afterload will also decrease, the pressure gradient for venous return is very small, in the order of 4–8 mmHg, so small increases in right atrial pressure will disproportionally decrease cardiac output unless preexisting volume overload prevents such decrease in venous return.

Patients subjected to hyperinflation and increased juxtacardiac pressure will experience a drop in biventricular volumes. Furthermore, some of these effects, including decreased vasomotor tone, may be affected by induction of anesthesia during initiation of mechanical ventilation. Lung inflation with volumes less than 10 mL per kilogram of body weight leads to vagal tone withdrawal and, consequently, an increase in heart rate. This is known as respiratory sinus arrhythmia. On the other hand, lung hyperinflation with volumes greater than 15 mL per kilogram of body weight leads to vagal tone activation and sympathetic tone withdrawal. In such cases, patients exhibit a decrease in blood pressure secondary to vasodilation from a decrease in systemic vasomotor tone. Many patients with LV failure are also hypervolemic owing to increased antidiuretic hormone activation. Thus, in volume overloaded patients with heart failure, initiation of mechanical ventilation and some degree of lung hyperinflation will decrease preload, cause vasodilation and, thereby, decrease intrathoracic blood volume. In those settings, the slight decrease in LV afterload may augment LV ejection keeping cardiac output constant despite a sustained increase in ITP. These are the reasons why patients with LV failure usually tolerate intubation and positive-pressure ventilation well without secondary post-intubation hypotension (Shepherd, 1981; Vieillard-Baron et al., 1985; Gomez et al., 2013).

### Ventilator weaning

Weaning patients from positive pressure ventilation results in several processes occurring simultaneously. First, respirations change from positive swings in ITP to negative swings in ITP. This must increase venous return and LV afterload. The combined effects of these two processes lead to an increase in intrathoracic blood volume. Second, metabolic demand increases as respiratory muscles resume their normal activity. Patients with increased work of breathing during weaning will experience sympathetic hyperactivation and catecholamine surge leading to increased heart rate and hypertension, which in turn will increase myocardial oxygen demand. If the work of breathing is excessive or LV functional reserve is too limited, then the patient will fail weaning. Weaning failure will manifest as acute cardiovascular collapse, hypotension, tachycardia, and even acute cardiogenic pulmonary edema. These hemodynamic changes can be ameliorated by restricting fluids, having the patients slightly hypovolemic and treating bronchospasm prior to extubation. Optimizing bronchodilation, avoiding volume overload and controlling afterload prior to weaning trials will facilitate liberation from mechanical ventilation (Jubran et al., 1998; Roche-Campo et al., 2018; Tavazzi, 2021).

### Chronic obstructive pulmonary disease

Patients with COPD can also have LV failure. Both COPD and obesity are associated with terminal airway collapse with increased shunt. COPD patients are more likely to experience hyperinflation owning to the increased airway resistance combined with increased lung compliance. These patients experience limitations in expiratory flow secondary to airway collapse. Both hyperinflation and dynamic hyperinflation can increase pulmonary vascular resistance and compress the heart within the cardiac fossa. The combined effects can worsen RV dysfunction by minimizing RV end-diastolic volume (EDV) while simultaneously increasing RV afterload. Patients with LV failure need a higher LV filling pressure and EDV to sustain LV stroke volume. Since hyperinflation compresses the heart, LV end-diastolic volume also decreases, and thus cardiac output often decreases during acute exacerbations of COPD in patients with LV dysfunction (Junhasavasdikul et al., 2018).

**FIGURE 2:**
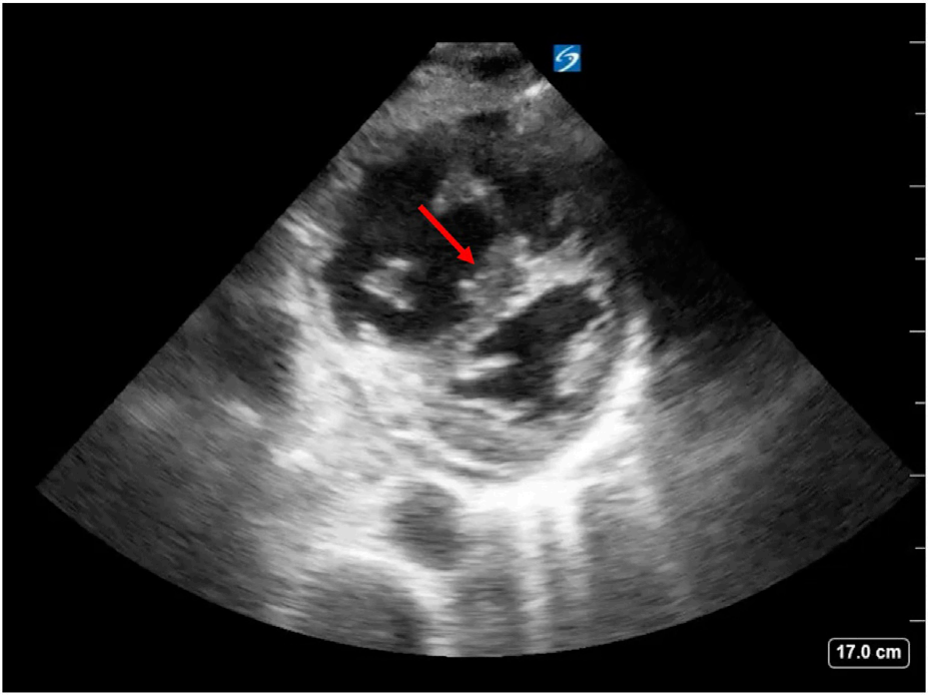
Negative swings in ITP, as seen with spontaneous inspiration in patients with COPD exacerbation, lead to increased RV end-diastolic volume. This effect in combination with increased RV afterload can cause RV dilation and intraventricular septal shift towards the LV leading to decreased LV end-diastolic compliance, decreased LV end-diastolic volume and decreased cardiac output. This is known as “D sign” on echocardiography (arrow). Image source: Dr. Christopher Schott, University of Pittsburgh Medical Center.

Commonly, patients with LV failure develop RV failure. In such cases, increases in transpulmonary pressure secondary to lung hyperinflation lead to increased RV afterload and decreased RV ejection and RV stroke volume leading to systemic hypotension. In these circumstances, patients may experience acute cor pulmonale and RV ischemia. COPD exacerbations can cause hypoxemia, hypercapnia and increased sympathetic tone. Passive increases in pulmonary artery pressure due to increased LV filling pressure rarely cause enough increases in pulmonary artery pressure to result in acute RV failure. These three mechanisms increase pulmonary vasomotor tone. Patients with COPD may also have chronic pulmonary hypertension leading to RV hypertrophy. Increasing RV afterload increases RV end-systolic volume and, by extension, RV EDV. Clinically, these patients may present with increased jugular venous distension during spontaneous inspiration (Kussmaul’s sign) and the shift in the intraventricular septum will decrease LV end diastolic compliance leading to decreased cardiac output (Figure 2) (Mooney et al., 2021). Limitations in diastolic volume caused by hyperinflation lead to a decrease in LV EDV and, consequently, impairment of LV stroke volume and its ability to respond to stress as hyperinflation causes a leftward move on the ventricular function curve. When hyperinflation is not present, the major limiting membrane defining absolute biventricular volume is the pericardium. Though minimally influencing diastolic filling under normal conditions, if the RV were to suddenly dilate as occurs with massive pulmonary emboli, pericardial pressure rapidly rises as the pericardium becomes the limiting membrane. In these conditions the increased RV EDV limits LV EDV. If sustained, this process rapidly results in cardiovascular collapse and hypotension. The immediate treatment of this process is to disconnect the patient from the ventilator to reverse hyperinflation and if hypotension persists, give a vasopressor to increase arterial pressure and, thereby, increase RV coronary perfusion pressure to allow for more effective RV ejection to a lower end-systolic volume. Importantly, giving fluids alone in this setting will likely worsen the physiologic state because the RV is unable to eject its EDV and more fluids further increase RV strain.

### Obstructive sleep apnea

Obstructive sleep apnea is characterized by intermitted upper airway obstruction leading to marked episodes of negative swings in ITP and decreases in arterial oxygenation. Waxing and waning respirations lead to hypoventilation and a drop in the P_a_CO_2_ beyond the apneic threshold. Furthermore, episodes of hypoxia and waking from sleep lead to sympathetic activation, which further increases LV afterload and contributes to an increase in myocardial oxygen demand. Patients with OSA have intermittent hypoxemia and formation of free radicals and inflammatory molecules that lead to vascular remodeling. The negative swings in ITP during OSA lead to a combined increase in LV afterload and systemic hypoxemia. Thus, at the time that LV wall stress is increasing, myocardial oxygen delivery is being compromised. Over months, these repetitive OSA events lead to the development of congestive heart failure and a downward shift in the ventricular function curve. Acutely, the negative swings in ITP, hypoxia, and sympathetic surge lead to increased LV afterload and reactive systemic hypertension leading to increased myocardial metabolic demand. Chronically, these factors worsen LV hypertrophy, which in turn affects LV diastolic function. OSA has been associated with LV remodeling and increased incidence of cardiovascular disease (Naughton and Bradley, 1998; Feihl and Broccard, 2009; Cundrle et al., 2019; Wahab et al., 2022).

Negative swings in ITP, hypoxia and repeated nocturnal awakening can be reversed using non-invasive positive pressure ventilation (NIPPV). Nocturnal CPAP or BiPAP have been shown to improve outcomes in patients with disordered sleep. Specifically, OSA patients with heart failure demonstrate improvement in daytime off-CPAP LV function if the CPAP treatment abolishes the obstructive breathing patterns (Khattak et al., 2018).

### Acute respiratory distress syndrome

Noncardiogenic pulmonary edema or ARDS is characterized by decreased lung volumes secondary to alveolar collapse and pulmonary congestion, increased dead space ventilation, hypoxemia and increased work of breathing. Often hypoxemia is not responsive to non-invasive ventilation and intubation with positive end-expiratory pressure (PEEP) is required to sustain gas exchange and minimize receptive alveolar collapse at end-expiration. The resultant higher mean and end-inspiratory airway pressure often further increase dead space ventilation. Dead space ventilation along with hypoxic pulmonary vasoconstriction secondary to alveolar collapse lead to increased pulmonary vascular resistance. This must impede RV ejection and increase RV EDV, which in turn decreases LV diastolic compliance by the process of ventricular interdependence. Indeed, Jardin et al. demonstrated many years ago that when ARDS patients were given high levels of PEEP their LV stroke volume decreased despite a high pulmonary artery occlusion pressure, used as a surrogate of LV EDV. However, when these same patients were fluid resuscitated to restore their LV EDV to their pre-PEEP levels, LV stroke volume also returned to its pre-PEEP value even though PEEP was still being used. This seminal work clearly demonstrated that lung hyperinflation and high levels of PEEP impair LV filling by decreasing LV diastolic compliance through the process of ventricular interdependence and increased juxtacardiac ITP. These points are described elsewhere in this series in greater detail (Jardin et al., 1991).

PEEP affects RV afterload by changing pulmonary vascular resistance though recruitment of collapsed alveoli (decreasing resistance) and overdistention (increasing resistance). This elusive “sweet spot” mean airway pressure that balances these two opposing effects is often equated to the point where PEEP maximizes lung compliance. Clinical studies suggest that the highest PEEP associated with the best value of lung compliance is also associated with better RV systolic function as measured using transesophageal echocardiography, least dead space ventilation, assessed as end-tidal CO_2_ and optimal cardiac output for a given blood volume. Titrating PEEP based on RV systolic function and lung compliance is an important technique to monitor for developing RV failure and preventing PEEP too low that would worsen alveolar collapse and hypoxia or PEEP too high that would worsen PVR and RV afterload and, consequently, worsen LV diastolic filling (Schmitt et al., 2001; Vieillard-Baron et al., 2016).

Other investigators looked at variables that are prominent in patients with ARDS that develop cor pulmonale. These investigators concluded that pneumonia being the etiology of ARDS, driving pressure, worsening P:F ratio and rising P_a_CO_2_ levels are all predictive of the development of cor pulmonale in ARDS. Clearly, all these effects are predictable based on the above logic. Driving pressure is a surrogate of lung stress due to its effect on hyperinflation and pulmonary vascular resistance. Likewise, higher P_a_CO_2_ levels are indicative of increased dead space and lead to pulmonary vasoconstriction and increased pulmonary vascular resistance. Mitigating these factors is critical to avoid right heart failure, RV dilation and, consequently, poor LV diastolic filling and low output heart failure (Mekontso Dessap et al., 2016).

## Conclusion

Heart-lung interactions play a crucial role in understanding and managing patients with left heart failure under most conditions independent of ventilatory mode.

## Abbreviations

ACPE, acute cardiogenic pulmonary edema; ARDS, acute respiratory distress syndrome; COPD, chronic obstructive pulmonary disease; CO, cardiac output; EDV, end-diastolic volume; ITP, intrathoracic pressure; LV, left ventricular; OSA, obstructive sleep apnea; PEEP, positive end-expiratory pressure; PVR, pulmonary vascular resistance; PPV, positive-pressure ventilation; RV, right ventricular; VR, venous return.
